# Cortical region-specific engraftment of embryonic stem cell-derived neural progenitor cells restores axonal sprouting to a subcortical target and achieves motor functional recovery in a mouse model of neonatal hypoxic-ischemic brain injury

**DOI:** 10.3389/fncel.2013.00128

**Published:** 2013-08-21

**Authors:** Mizuya Shinoyama, Makoto Ideguchi, Hiroyuki Kida, Koji Kajiwara, Yoshiteru Kagawa, Yoshihiko Maeda, Sadahiro Nomura, Michiyasu Suzuki

**Affiliations:** ^1^Department of Neurosurgery, Yamaguchi University School of MedicineUbe, Japan; ^2^Department of Systems Neuroscience, Yamaguchi University School of MedicineUbe, Japan; ^3^Department of Neurosurgery, Ube Nishi Rehabilitation HospitalUbe, Japan

**Keywords:** hypoxic-ischemic encephalopathy, embryonic stem cell, transplantation, regeneration, integration, Ctip2, motor function

## Abstract

Hypoxic-ischemic encephalopathy (HIE) at birth could cause cerebral palsy (CP), mental retardation, and epilepsy, which last throughout the individual's lifetime. However, few restorative treatments for ischemic tissue are currently available. Cell replacement therapy offers the potential to rescue brain damage caused by HI and to restore motor function. In the present study, we evaluated the ability of embryonic stem cell-derived neural progenitor cells (ES-NPCs) to become cortical deep layer neurons, to restore the neural network, and to repair brain damage in an HIE mouse model. ES cells stably expressing the reporter gene GFP are induced to a neural precursor state by stromal cell co-culture. Forty-hours after the induction of HIE, animals were grafted with ES-NPCs targeting the deep layer of the motor cortex in the ischemic brain. Motor function was evaluated 3 weeks after transplantation. Immunohistochemistry and neuroanatomical tracing with GFP were used to analyze neuronal differentiation and axonal sprouting. ES-NPCs could differentiate to cortical neurons with pyramidal morphology and expressed the deep layer-specific marker, Ctip2. The graft showed good survival and an appropriate innervation pattern via axonal sprouting from engrafted cells in the ischemic brain. The motor functions of the transplanted HIE mice also improved significantly compared to the sham-transplanted group. These findings suggest that cortical region specific engraftment of preconditioned cortical precursor cells could support motor functional recovery in the HIE model. It is not clear whether this is a direct effect of the engrafted cells or due to neurotrophic factors produced by these cells. These results suggest that cortical region-specific NPC engraftment is a promising therapeutic approach for brain repair.

## Introduction

Hypoxic-ischemic encephalopathy (HIE) represents a major cause of brain damage in the fetus and newborn infants, occurring in about 20 per 1000 term live birth, and in nearly 60% of very low birth weight newborns (MacDonald et al., 1980; Gunn, 2000). It is suggested that up to 25% of the survivors have permanent and varied neurological deficits, including cerebral palsy (CP), mental retardation, learning disability, and chronic epilepsy (Vannucci, 2000; Volpe, 2001; Ferriero, 2004). These deficits can severely impair the patients' ability in their daily life (ADL) throughout their lifetime (Perlman, 2006).

Hypoxic ischemia (HI) stress triggers neuronal and glial injury leading to necrosis secondary to cellular edema, lysis, and the resulting apoptosis in delayed cellular injury (Delivoria-Papadopoulos and Mishra, 1998; Nakajima et al., 2000; Northington et al., 2001). These cellular injuries tended to occur in the subplate neurons and resulted in periventricular leukomalacia (PVL) due to immaturity of the cerebral vasculature or its vulnerability (Volpe, 2001; McQuillen et al., 2003). The cortex is partitioned into distinct regions that serve different functions, such as vision, touch, hearing, and movement. Each of these areas is composed of six layers of glutamatergic pyramidal neurons with distinct firing patterns, specific axonal and dendritic projection patterns (Connors and Gutnick, 1990; Tseng and Prince, 1993), and unique gene expression profiles including bHLH transcriptional factors, such as neuroD, neuroD2, and Math2, which are all expressed in newborn cortical projection neurons at least transiently, and also two T-domain transcriptional factors, Tbr2 and Tbr1, are expressed sequentially in many projection neurons during differentiation (Hevner, 2006). These neurons degenerate in amyotrophic lateral sclerosis (ALS) which mainly affects late onset motor neurons (Boillee et al., 2006), in spinal cord injury which induces apoptosis of cortical projection neurons (Hains et al., 2003), and also in HIE which induces cortical laminar necrosis, predominantly in pyramidal cell layers III or V (Armstrong et al., 2007). With the exception of hypothermia, there is no specific effective treatment which could rescue HIE patients (Gluckman et al., 2005; Perlman, 2006). An effective therapy is needed to treat these patients.

Cell replacement therapy offers the prospect to rescue damaged tissue, to replace lost cells, and restore neurological function after HI encephalopathy. Embryonic stem (ES) cells possess attractive features as a limitless cell source to create the myriad of cell types found in the body. The finding that neurons can be generated from ES cells has led to the expectation that neurological pathologies, such as Parkinson's disease (PD), ALS, stroke, or spinal cord injury will be treatable using cell-based transplantation strategies (Muotri and Gage, 2006; Suzuki and Svendsen, 2008). Neural stem cell-based therapy is also an attractive novel therapy for neonatal HIE, but not much research as been done in this area (Daadi et al., 2010).

One would hypothesize that when using a cell-based therapy for HIE, motor functional recovery could be achieved with the replacement of injured cortex by implantation of pyramidal neuronal progenitor cells and that the cortical area-specific axonal projections to the subcortical area would recapitulate the normal adult structure.

In the present study, we evaluated functional recovery after implantation of ES cell-derived neuronal progenitor cells (ES-NPCs) into a neonatal HIE model. We also investigated whether the implanted cells can generate deep layer cortex-specific pyramidal neurons and recapitulate an area-specific neuronal network based on axonal projections from the implanted cells.

## Materials and methods

### Animals and experimental groups

Pregnant mice were purchased from Japan SLC (Shizuoka, Japan). Two day-old neonatal ICR mouse pups were randomly assigned to the following four groups: (1) Naïve control group (no operative procedure), (2) HI control group (HI only, no transplantation), (3) vehicle-transplantation group (HI and vehicle-transplantation), (4) Transplantation group (HI and NPC-transplantation). Animal experiments were performed in accordance with the guidelines of Yamaguchi University and *the NIH Guide for the Care and Use of Laboratory Animals.*

### Cell preparation

The F7 cell line, which was generated from the ES cell line E14Tg2a.4 and contained the inserted green fluorescent protein (GFP) expressing gene, was cultured as previously described (Ideguchi et al., 2008). Briefly, cells were plated at low density, 10^6^ per 10 cm dish coated with gelatin (Millipore Bioscience Research Reagents; ES-006-B), for 10 days in “Maintenance medium” consisting of Glasgow modified Eagle's medium (GMEM; Sigma) supplemented with 10% FBS (Hyclone), 2 mM L-glutamine (Gibco-Invitrogen), 0.1 mM non-essential amino acid (NEAA;Gibco-Invitrogen), 1 mM sodium pyruvate (Sigma), 0.1 mM 2-mercaptoethanol (Sigma), and 2000 units/ml leukemia inhibitory factor (LIF; Esgro, Chemicon) under Geneticin selection (1.5 μg/ml; Invitrogen). ES cells were induced to a neural precursor state by co-culture with the mouse stromal cell line MS5 (Kirin Pharma) (Barberi et al., 2003). “MS5 medium” consisted of α MEN (Gibco) supplemented with 10% fetal bovine serum (FBS; Sigma) for 7 days. Briefly, a confluent plate of MS5 cells were treated with mitomycin C (2 μg/ml) for 2 h, washed, dissociated, and plated onto gelatin-coated 10 cm dishes. The next day, 10^4^ ESCs were added and cultured in “Differentiation medium” consisting of GMEM (Gibco-Invitrogen) supplemented with 10% Knockout Serum Replacement (KSR; Gibco-Invitrogen), 2 mM L-glutamine, 0.1 mM NEAA, 1 mM sodium pyruvate, and 0.1 mM 2-mercaptoethanol. FGF2 (fibroblast growth factor 2; R&D Systems) at 20 ng/ml was added for the last 2 days and resulted in increased numbers of cortical pyramidal neurons. ES-NPC colonies were removed by incubation in Ca2^2+^, Mg^2+^-free HBSS (15 mM HEPES) for 30–45 min. These ES-NPC colonies were dissociated in papain (Worthington Biochemicals) for 20 min at 35°C and triturated into a single-cell suspension. The dissociated ES-NPCs were either plated for immediate immunostaining or further differentiated into mature neural cells after plating at 1.5 × 10^5^ cells in 500 μ l of NB media (Neurobasal and B27; Invitrogen) in each well. Eight-well chamber slides (Falcon; BD Biosciences Discovery Labware) were coated with polyornithine (100 ug/ml) followed by fibronectin (100 ug/ml) (Millipore) before use. The ES-NPCs were allowed to differentiate for 7 days and then fixed in 4% paraformaldehyde.

### Hypoxia-ischemia model

The model used in this study was based on the Rice-Vannucci model (Vannucci and Vannucci, 1997). Pups were housed with the dam under a 12:12 h light-dark cycle, with food and water available *ad libitum* throughout the studies. On postnatal day 2, the pups were anesthetized by inhalation with halothane (induction at 4% and then maintained at 1.0–1.5%), 70% nitrous oxide, and 30% oxygen. The mice were incised at the midline in a linear fashion from the infragnathia to the sternum. Then, the right common carotid artery (CCA) was exposed and ligated with 10-0 nylon thread at two points. Between the ligated points, the CCA was cut after treatment with a bipolar coagulator®. After the operation, the pups were returned to the holding container which was maintained at a temperature of 37°C and they were allowed to recover from anesthesia for 1–2 h. Then, they were placed in a humidified chamber in a 37°C water bath perfused with a hypoxic gas mixture (8% oxygen, 92% nitrogen) for 20 min. All surviving pups were returned to their dams after hypoxic exposure. From the time of the operation to the hypoxia treatment, the pups were continuously kept at a temperature of 37°C (HI model mouse group; *n* = 12, control normal mouse group;*n* = 12).

### TTC-labeling method

At 24 h postoperative, the mice were anesthetized and sacrificed by rapid decapitation. One mm thick coronal brains sections were cut using a mouse brain matrix (Activation Systems, Inc.), and sections were immersed in 2% 2,3,5-triphenyltetrazolium chloride (TTC; Sigma Chemical Co) in 0.9% saline solution, as described (Bederson et al., 1986).

### Transplantation

Dissociated day 7-differentiated ES-NPCs were transplanted into the right cerebral cortex of P4 neonatal mice. Two days after the induction of HI, animals were placed in a stereotaxic apparatus with a neonatal mouse adaptor. Pups were anesthetized systemically by inhalation of 4.0–1.0% halothane. After a midline linear skin incision was made, the skull bregma was determined and a single cell suspension (50,000 cells/μ L) of ES-NPCs was transplanted using a fine tipped, sterile glass pipette into 4 sites (1 μ L/site) in the right ischemic hemisphere targeting to the motor cortex [from the bregma: (1) antero-posterior (AP) 0 mm, lateral (L) 0.5 mm; vertical (V) 0.5 mm; (2) AP 0 mm, L 1.5 mm, V 0.5 mm; (3) AP 1.0 mm, L 0.5 mm, V 0.5 mm; (4) AP 1.0 mm, L 1.5 mm, V 0.5 mm] over several minutes (*n* = 10). Sham transplantation was performed as a control using the surgical procedure itself (*n* = 10). The skin was closed with tissue adhesive (Vetbond 3M) and the pups were resuscitated under a warming lamp. Three weeks after transplantation, mice were perfused transcardially. Brains were removed and sectioned at 60 um thickness. Free-floating sections were immunostained with the indicated primary antibodies and appropriate secondary antibodies, as described below. DAB (Sigma, D5905) enhancement was used to visualize the graft of the GFP-positive cells. The histological images were acquired on a Keyence BZ-9000 fluorescence microscope (Keyence, Osaka). The number of CTIP2-positive cells in five arbitrary squares (200 × 200 μ m) per randomly selected region of layer V area in each section was counted in every third section, and we calculated the average number of positive cells in the unit area (4.0 × 10^4^ μ m^2^). A total of at least 40 areas were counted per data point. Image joint and dynamic cell count software (Keyence, Osaka) was used to measure the cortex area, respectively as described previously (Kida et al., 2013).

### Immunohistochemistry

After fixation in 4% paraformaldehyde, followed by permeabilization and blocking with 0.3% Triton X-100 and 2% skim milk for cell culture or 5% normal goat serum for brain sections, cells or sections were incubated at 4°C overnight in primary antibodies. Primary antibodies included GFP (1:2000; final concentration 1.0 μg/ml in DAB staining, 1:200; final concentration 10 μg/ml in fluorescent staining, Molecular Probe; A11122), Oct3/4 (1:100; final concentration 2.0 μg/ml, Santa Cruz Biotechnology; SC-8628), Nestin (1:200; final concentration 2.5 μg/ml, Millipore Biosciences Pharmingen; 556309), β-Tubulin-III (1:400; final concentration 2.5 μg/ml, Covance Research Products; PRB-435P), Map2ab (1:200; final concentration 10 μg/ml, Sigma-Aldrich; M1406), GFAP (1:2000, Millipore Biosciences Pharmingen; AB5804), Ctip2 (1:500; final concentration 2.0 μg/ml, Abcam; ab18465), Cux1 (1:200; final concentration 1.0 μg/ml, Santa Cruz Biotechnology; sc-13024), Otx1 (1:200, Abnova; H00005013-A01), Vesicular Glutamate Transporter 1 (VGLUT1) (1:500; final concentration 2.0 μg/ml), Chemicon; MAB5502 and GABA (1:2000, ImmunoStar; 20094). Appropriate cyanin-3(Cy3)-, FITC-, cyanin-5(Cy5)-labeled secondary antibodies (Jackson Immunoresearch Laboratories, Inc., West Grove, PA) were used at room temperature for 2 h. For nuclear staining, 4',6-diamidino-2-phenylindole (DAPI) (200 ng/mL) was added to the final wash. The immunoreactive cells were detected using either a laser confocal microscope (Zeiss LSM5) or a fluorescent scope (BZ-8000; Keyence, Osaka, Japan). The cells in 10 fields were counted in each of 4–6 independent experiments. The total number of cells was evaluated by counting DAPI-positive nuclei. Because sometimes the vendor does not provide the information about concentrations of optimal-working antibody, we first determined the appropriate concentration. For initial experiments, we tried dilutions ranging from 1:50 to 1:2000 for immunohistochemical applications. And then, we determined most suitable concentration or dilution ratio. The detailed information of primary antibodies we used in this study was shown in Table 1. Regarding tissue type control, we referred product sheet information and references that were listed in it, and then selected the optimal positive and negative control. Each positive and negative control we used was listed in Table 2.

**Table 1:** **Antibodies information used in this study**.

**Antibody**	**Isotype**	**Concentration**		**Source/Catalog number**	**Host**	**Immunogen**
		**Primary**	**Final**			
GFP	IgG2a	2.0 mg/ml	10 μg/ml (in fluorescent)	Molecular Probe/A11122	Mouse	Green fluorescent protein
			1.0 μg/ml (in DAB)			
Oct3/4	Polyclonal	200 μg/ml	2.0 μg/ml	Santa Cruz Biotechnology/SC-8628	Goat	Human Oct-3A/4
Nestin	IgG1	0.5 mg/ml	2.5 μg/ml	Millipore Biosciences Pharmingen/556309	Mouse	E15 Rat spinal cord extracts
β-Tubulin-III	Polyclonal	1.0 mg/ml	2.5 μg/ml	Covance Research Products/PRB-435P	Rabbit	Rat brain microtubules
Map2ab	IgG1	2.0 mg/ml	10 μg/ml	Sigma-Aldrich/M1406	Mouse	Bovine MAP2
GFAP	Polyclonal	Not described	1:2000 dilution	Millipore Biosciences Pharmingen/AB5804	Rabbit	Bovine GFAP
Ctip2	IgG2a	1.0 mg/ml	2.0 μg/ml	Abcam/ab18465	Rat	Fusion protein (amino acids of human CTIP2)
Cux1	Polyclonal	2.0 μg/ml	1.0 μg/ml	Santa Cruz Biotechnology/sc-13024	Rabbit	Mouse origin CCAAT displacement protein (CDP)
Otx1	Polyclonal	Not described	1:200 dilution	Abnova/H00005013-A01	Mouse	Human OTX1 partial recombinant protein
VGLUT1	IgG1	1.0 mg/mL	2.0 μg/ml	Chemicon/MAB5502	Mouse	Rat vesicular glutamate transporter (VGLUT) 1
GABA	Polyclonal	Not described	1:2000 dilution	ImmunoStar/20094	Rabbit	γ (gamma)-aminobutyric acid (GABA) coupled to bovine serum albumin (BSA)

**Table 2:** **Tissue type controls used in this study**.

**Antibody**	**Positive control**	**Negative control**
GFP	GFP stably expressed ES cells that were constructed from E14Tg2a.4 (Ideguchi et al., 2010)	Undifferentiated mouse ES cells line, E14Tg2a.
Oct3/4	Day 3.5 blastocysts that were extracted from the uteri of pregnant mouse female	Postnatal day 1 (P1) mouse brain
Nestin	Embryonic day 14 (E14) mouse brain	Undifferentiated mouse ES cells line, E14Tg2a Adult mouse brain
β-Tubulin-III	Postnatal day 1 (P1) mouse brain	Undifferentiated mouse ES cells line, E14Tg2a
Map2ab	Adult mouse brain	Undifferentiated mouse ES cells line, E14Tg2a
GFAP	Adult mouse brain	Undifferentiated mouse ES cells line, E14Tg2a Mature neurons of adult mouse brain as internal control
Ctip2	Postnatal day 1 (P1) mouse brain	Undifferentiated mouse ES cells line, E14Tg2a
Cux1	Postnatal day 1 (P1) mouse brain	Undifferentiated mouse ES cells line, E14Tg2a
Otx1	Embryonic day 14 (E14) mouse brain	Undifferentiated mouse ES cells line, E14Tg2a
VGLUT1	Adult mouse brain	Undifferentiated mouse ES cells line, E14Tg2a
GABA	Adult mouse brain	Undifferentiated mouse ES cells line, E14Tg2a

### Functional analysis

To evaluate balance and motor coordination, mice were tested at 3 weeks after transplantation for their sensorimotor skills in both the Rotarod and Beam walking tests, as previously reported (Daadi et al., 2009). Briefly, in the Rotarod test, the mice were placed onto a horizontal rotating rod at 4 rpm (Model ENV-576M; Med Associates, St. Albans, VT, USA), and then the treadmill was accelerated from 4 to 40 rpm over the course of a 5-min trial. A single test lasted from the time that the mouse was placed on the rotating rod until it fell-off or until 5 min had elapsed. The mice rested in their home cages for 20–30 min between each trial. The average time on the rotating rod is presented. In the Beam walking test, the beam was 0.6 cm wide and 120 cm in length and was suspended about 60 cm above foam pads. The average score (total time spent on beam walking divided by 5 trials) was calculated for each animal.

### Statistical analysis

All values were expressed as mean ± SD. Statistical comparisons among each group were determined using a commercially available software package (JMP 9.0, SAS Institute Inc., Cary, NC). Data were tested by the Wilcoxon signed-rank test. *P* < 0.05 was considered statistically significant.

## Results

### Characterization of hypoxia-ischemia injury

**Figure 1:**
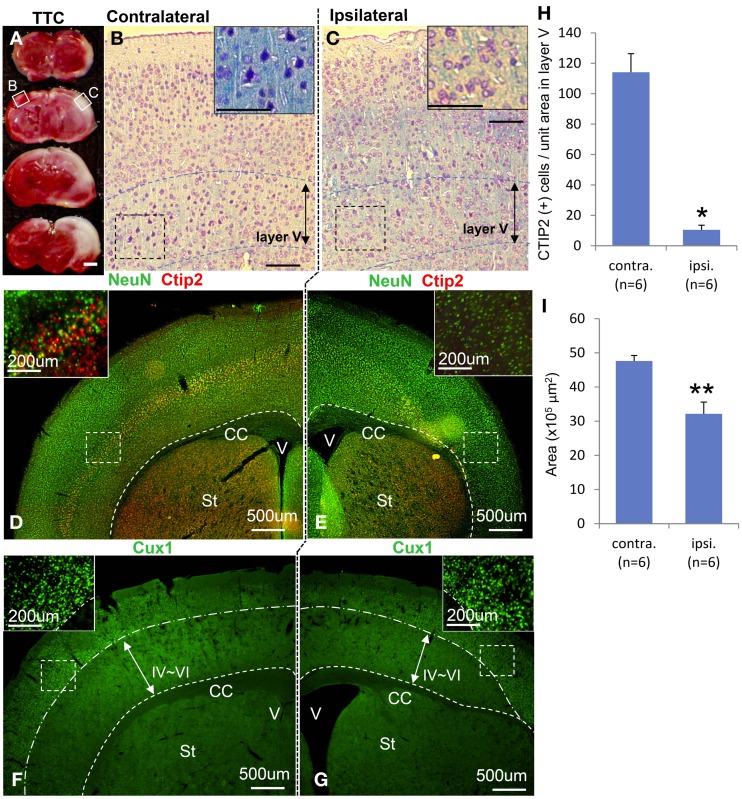
**Characteristics of hypoxia-ischemia encephalopathy and motor function.** Ischemic lesion of cortex in TTC staining 3 weeks after HI exposure is shown in **(A)** with an unstained area. Kluver–Barrela stained sections show layer V pyramidal neurons in the contralateral cortex **(B)** that is presented as a square area with solid line in **(A)**. Magnified insert in **(B)** shows monopolar and pyramidal-shaped neurons. On the other hand, layer V pyramidal-shaped neurons in the ipsilateral cortex are mostly absent (**C** and magnified insert) as shown in the square area with a solid line in **(A)**. Immunostaining shows the mature neuronal marker NeuN, deep layer marker Ctip2 (**D** and magnified insert), and superficial marker Cux1 (**F** and magnified insert). Ctip2-positive neurons in the ipsilateral cortical area dramatically decreased (**E** and magnified insert), while Cux1-positive cells in the superficial area are comparatively preserved (**G** and magnified insert). **(H)** CTIP2 positive cells of unit-squared area (200 × 200 μ m) in randomly selected area in layer V cortex are represented by ipsilateral/contralateral hemisphere (^*^P < 0.0005). **(I)** The area of cortex is represented by ipsilateral/contralateral hemisphere (^**^P < 0.05). Scale bar: **(A)** 1000 μm; **(B,C)** 100 μm; **(D–G)** 500 μm; (**D–G**, magnified insert) 200 μm; CC: corpus callosum, V: ventricle, St: striatum.

**Figure 2:**
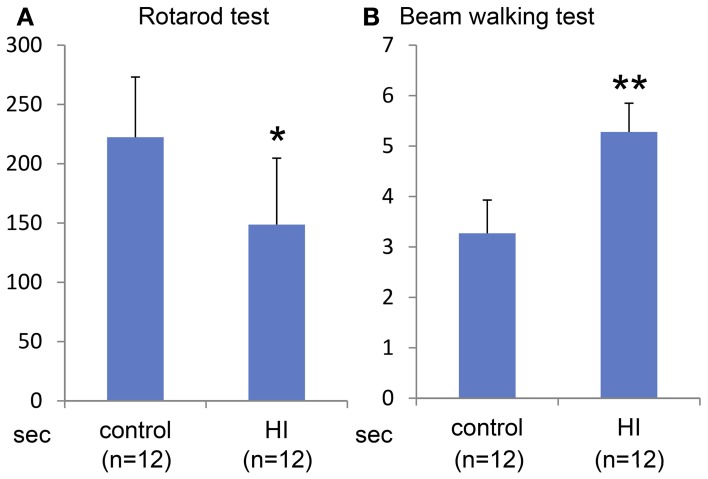
**Behavioral evaluation at 3 weeks after HI insult.** Rotarod test **(A)** and Beam walking test **(B)** show that somatomotor function disorder in the HIE model mice compared to control group with statistical significance (^*^P < 0.05, ^**^P < 0.001).

Macroscopically, the lateral side of the ischemic cerebral hemisphere turned white clearly indicating ischemia and edema at 24 h after the HI insult (Data not shown). TTC staining showed an ischemic lesion of the cortex in the same region (Figure 1A). Microscopic examination of the Kluver–Barrela staining showed loss of pyramidal-shaped neurons in the deep layer (Figure 1C and magnified photograph) when compared to the contralateral cortex (Figure 1B and magnified photograph). In addition, Ctip2 immunostaining, which is a specific marker of layers V and VI, demonstrated that the number of positive neurons in layers V–VI (deep layer) was significantly reduced by almost 90% compared to the contralateral side (Figures 1D,E,H, *P* = 0.0002). The thickness of the cortex of the HIE model; 3.44 × 10^6^ μ m^2^, was also significantly attenuated compared to the contralateral side; 4.76 × 10^6^ μ m^2^, (Figure 1I, *P* = 0.036). These findings are in contrast to the preservation of Cux1-positive superficial layer neurons (Figures 1D,E,F,G and magnified photographs). An assessment of the motor function at 3 weeks after HI insult showed that the HIE model mice had lower scores in the Rotarod (Figure 2A, *P* = 0.012) and Beam walking tests (Figure 2B, *P* = 0.0002).

### *In vivo* neuronal differentiation of ES cells toward deep layer-specific neurons

**Figure 3:**
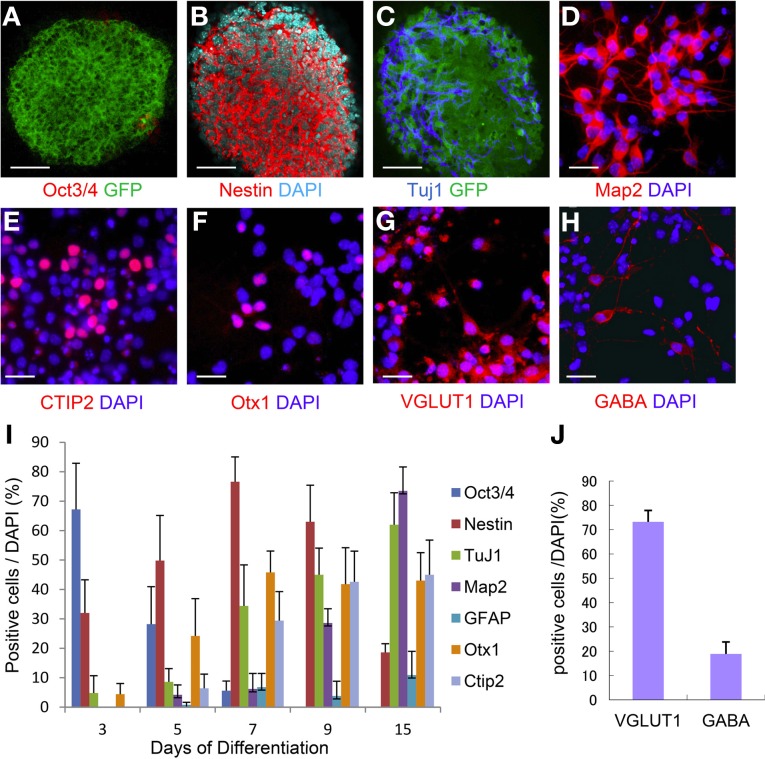
**Time course of ES cell neural induction with MS5 co-culture.** Colonies of ES cells grown on MS5 cells for 7 days were immunostained for Oct3/4 **(A)** and GFP, nestin **(B)**, Tuj1**(C)**, and GFP. Immunostaining of day 7 MS5-NPCs after an additional 8 days of differentiation into mature cells on polyornithine/fibronectin (OF)-coated slides, Map2ab **(D)**, Ctip2 **(E)**, Otx1 **(F)**, VGLUT1 **(G)**, and GABA **(H)**. **(I)** Time course of immunostaining for Oct3/4- Nestin-, TuJ1-, Map2ab-, GFAP-, Otx1-, and Ctip2-positive cells as a percentage of total DAPI-stained cells. **(J)** Frequency distribution of glutamatergic and GABAergic neuronal markers as a percentage of total DAPI stained cells after 15 days of neuronal differentiation. Scale bar; **(A–C)**, 50 um; **(D–H)**, 25 um.

ES cells were placed on a confluent layer of MS5 stromal cells for neuronal differentiation as done previously (Barberi et al., 2003; Ideguchi et al., 2010). The neural differentiation profile is shown in Figure 3I. After 7 days of co-culture, ES cell-derived colonies were observed, in which the majority (76%) of the cells expressed nestin that is known as a marker of immature neural cells (Figures 3B,I) whereas Oct3/4 positive-cells were hardly expressed, and subsequently were not detectable by day 15 (Figures 3A,I). Approximately 30% of the cells within the colonies expressed TuJ1 (Figures 3C,I), an early neuronal marker at day 7. Map2ab-positive mature neurons were less abundant than other immature neuronal cells at day 7, but increased with time until day 15 (Figure 3I). The number of GFAP-positive glial cells increased with time, but was less than 10% of the total cells (Figure 3I).

We next examined the potential of these MS5 co-cultured ES-NPCs to differentiate into more specific neurons. Day 7 ES cell-derived colonies were detached from the MS5 feeder layer, dissociated into single cells, and then further differentiated for an additional 8 days on coated slides in Neurobasal medium before immunohistochemical analysis. Map2ab expression on day 15 was increased up to ~70% in all cells (Figure 3D). Cell staining with deep cortical layer-specific markers, Otx1 and Ctip2, increased significantly between days 5 and 9, and afterward 45–50% of all cells expressed these genes on day 15 (Figures 3E,F,I). On day 15, ~74% of all cells were glutamatergic and 20% were GABAergic neurons, which was similar to the amounts present in the normal cortex (Figures 3G,H,J).

### Engrafted ES-NPCs expressed mature and deep layer-specific neuronal markers

**Figure 4:**
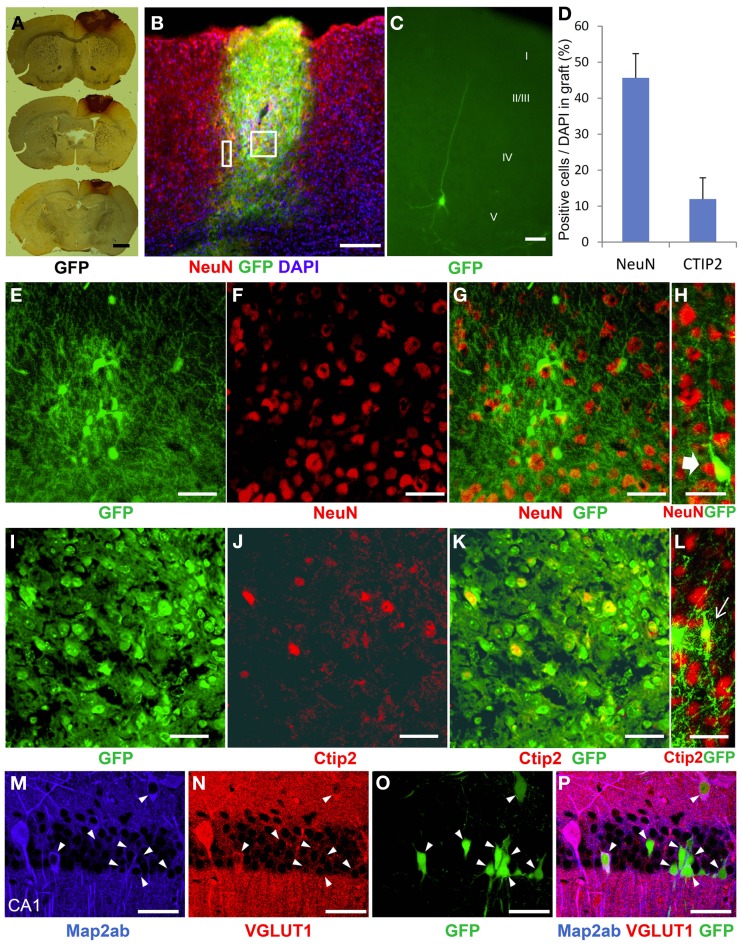
**Neurological differentiation of ES-NPCs 3 weeks after transplantation into HI-injured brain. (A)** DAB staining for GFP antigen showed that the survived graft located mainly in the cortex. **(B)** GFP-positive survived graft cells expressed NeuN, a mature neuronal marker, in the cortex and partially in the corpus callosum. The square area in **(B)** shows **(E–G)** and **(I–K)**, and the rectangle area shows **(H)** and **(L)**. **(C)** Some NPCs migrated into the cortex from the graft and differentiated into pyramidal-shaped neurons with apical dendrites. Additional dendrites and axons were observed in the layer V cortical area. **(D)** Frequently distribution of neuronal markers, NeuN and Ctip2, as a percentage of DAPI in the graft. Core region of graft showed co-localization of NeuN **(F)** or Ctip2 (**J**) and GFP (**E,I,G,K**). Some NeuN-positive **(H)** or Ctip2-positive cells **(L)** demonstrate monopolar morphology. **(M–P)** Migrated or leaked NPCs into the hippocampus at transplantation differentiated to CA1 pyramidal neurons with apical dendrites and also expressed the Map2ab neuronal marker and glutamate (arrow heads). Scale bar; **(A)** 1000 um, **(B)** 200 um, **(C)** 20 um, **(E–P)** 50 um.

To characterize the identity of the cells in the ES cell-derived graft, we performed immunohistochemical staining in the brains at 3 weeks after transplantation. GFP-DAB staining showed apparent engrafted cells in the injured cortex (Figure 4A). The graft in the HI-injured brain expressed not only the mature neuronal marker, NeuN, but also the deep layer cortical-neuronal marker, Ctip2 (Figures 4B,E–G,I–K). Some of the NeuN- positive and also the Ctip2-positive cells showed monopolar morphology suggesting that they were differentiating into deep layer neurons (Figures 4H,L). ES-NPCs migrated into the deep layer of the cortex from the core region of the GFP-positive graft and appeared differentiated to pyramidal neurons morphologically. These differentiated cells possessed apical dendrites extending toward the pial surface and axons descending into the subcortical area (Figure 4C). Regarding the composition in the graft, nearly 45% of all cells were NeuN positive and 12% were Ctip2 positive (Figure 4D). These data showed that ES-NPCs, even if implanted into an impaired cortex, possess the ability to differentiate into cortical deep layer pyramidal neurons based on both their morphology and also on their immunohistochemical staining. Interestingly, a portion of the cells that were unintentionally injected and/or leaked into the hippocampus survived in the CA1 pyramidal cell layer, and differentiated into CA1 pyramidal neurons with apical dendrites extended into the molecular cell layer. These cells in the hippocampus also stained with a mature neuronal marker and produced glutamate (Figures 4M–P). These findings show that deep layer neuron-specified NPCs seems to migrate into their preferred location, and then differentiate into region-specific and -functional mature neurons.

### Engrafted neurons project axons to subcortical brain locations

**Figure 5:**
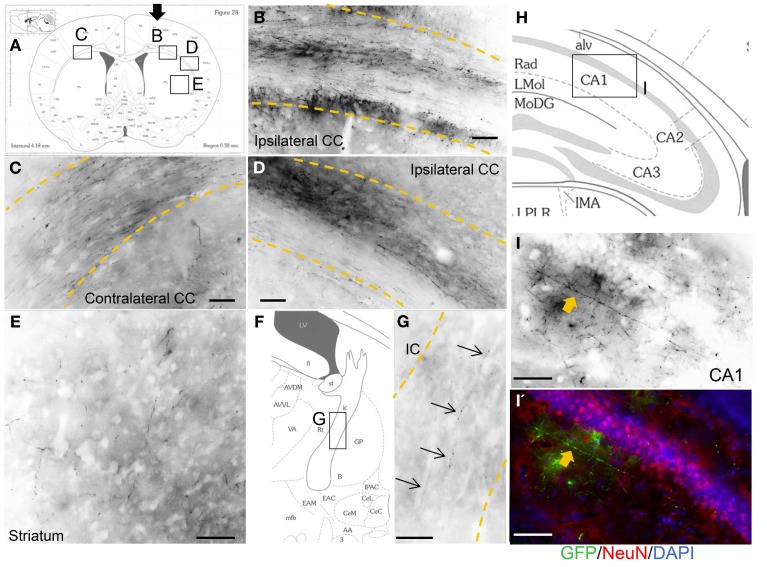
**Axonal outgrowth from the motor cortex transplant.** Schematic representations determined from the mouse brain of each photograph (Franklin and Paxinos, 2008), **(A)** coronal section, **(F)** magnified coronal section centering around the internal capsule, and **(H)** magnified coronal section centering around the hippocampus. Black arrow in **(A)** shows cells injection site. GFP-positive axons from the graft entered and extended within the corpus callosum (CC) just below the injection site **(B)**, lateral side **(D)**, striatum **(E)**, internal capsule (IC) (**G**, thin arrows), and also contralateral CC (**C**). (**I, I'**) Axon in the CA1 area in the hippocampus (thick arrows). Scale bar; (**B–E, G**) 100 um, (**I, I'**) 50 um.

In most of the grafted animals, we found GFP-positive axonal sprouting from the graft core into the subcortical target area. Axons from the sensorimotor cortex area extended into the ipsilateral side-corpus callosum (Figure 5B), further lateral side (Figure 5D), striatum (Figure 5E), internal capsule (Figure 5G), and contralateral corpus callosum (Figure 5C). Furthermore, a few GFP-positive axons extended into the molecular layer in the hippocampal CA1 region (Figures 5I,I′). Schematic representations determined from the mouse brain of each photograph (Franklin and Paxinos, 2008), (Figure 5A) coronal section, (Figure 5F) magnified coronal section centering around the internal capsule, and (Figure 5H) magnified coronal section centering around the hippocampus.

### Transplantation of ES-NPCs improved motor function

**Figure 6:**
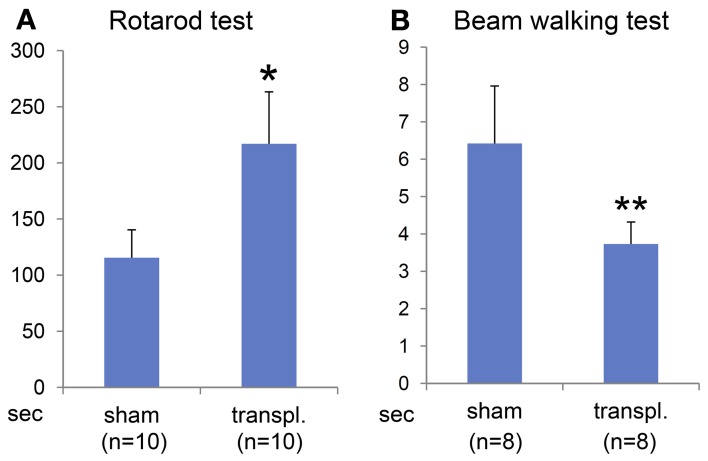
**Behavioral evaluation 3 weeks after NPCs transplantation into HI-injured brain.** Rotarod test **(A)** and Beam walking test **(B)** showed somatomotor functional recovery in the HIE model mice when compared to sham transplantation group with statistical significance (^*^P < 0.001, ^**^P < 0.005).

To determine the efficacy of the ES-NPCs engraftment for functional recovery, the sensorimotor skills of the animals were evaluated using 2 neurobehavioral tests 3 weeks after transplantation. Our results showed that after 3 weeks, the transplanted HIE mice significantly improved in the use of their limbs in both the Rotarod test (Figure 6A, *P* = 0.0008) and the Beam walking test (Figure 6B, *P* = 0.0014) when compared to sham-transplanted mice.

## Discussion

Our goal was to establish a therapeutic strategy for hypoxia-ischemia encephalopathy (HIE) using NPCs grafted into the brains of injured mice. We previously reported that ES cell-derived NPCs engrafted into the neonatal cortex and generated pyramidal shaped neurons which demonstrated axonal sprouting into appropriate subcortical-specific areas, thus, recapitulating the normal brain anatomy (Ideguchi et al., 2010). ES-NPCs possesses the ability to differentiate into dorsal forebrain neurons based on their gene expression profile (Ideguchi et al., 2010). Some reports demonstrated that pluripotent stem cell-derived neurons grew and developed neuronal connectivity with either adult athymic, immunosuppressed, damaged, or normal neonatal rodent brain (Gaillard et al., 2007; Thompson et al., 2009; Gaillard and Jaber, 2011; Denham et al., 2012; Steinbeck et al., 2012). Daadi et al. demonstrated that human neural stem cells which engrafted into the ischemic-injured adult brain had enhanced axonal sprouting into subcortical target areas, and increased expression of genes related to neurogenesis, resulting in motor functional recovery of the animals (Daadi et al., 2010). However, they emphasized that this functional recovery was not attributable to reconstruction via engrafted NSCs with host brain but due to neurotrophic support from those cells. In this study, we demonstrated axonal sprouting from engrafted cells into the subcortical target area. Because the location of the transplanted graft was chiefly in the motor cortex, the axons extended into the pyramidal tract-related area, i.e., corpus callosum, striatum, and internal capsule in accordance with normal axonal network anatomy. Thus, axonal rewiring can be achieved in not only the normal brain but also in the ischemic-insult brain environment. Moreover, we have shown that transplanted ES-NPCs expressed not only the mature neuronal marker, NeuN, but also another neuronal marker, Ctip2 which was expressed in the deep-layer cortical and axonal projecting neurons. Ctip2 expression in the graft supports the interpretation that some component of the graft can extend axons toward the subcortical region-specific target area. It is important to establish differentiation procedures aimed at generating cortical progenitors with different laminar identities to innervate appropriate target areas after transplantation. In our HIE insult model, we used animals with an injury to the deep layer cortex. The ES-NPCs used in this study are programmed to generate deep layer cortical-projection neurons, which was compatible with transplantation as a cell replacement procedure.

With one exception, the expression of a deep layer-specific marker, such as either Ctip2, Tbr1, Otx1, etc., had not been confirmed in the graft whereas a mature neuronal marker was identified (Daadi et al., 2010; Gaillard and Jaber, 2011; Steinbeck et al., 2012). Denham et al. demonstrated not only a mature neuronal marker but also Ctip2 and Tbr1 expression in the reporter gene-GFP positive graft. The graft location, however, was near the pial surface which was an incorrect area for Ctip2 and Tbr1 localization (Denham et al., 2012). In our study, some migrated NPCs from the graft generated pyramidal-shaped neurons that possessed extended apical dendrites and axonal projections, which have neuronal morphology similar to that found in the deep layer (Figure 4C). These novel findings that were based on appropriate engraftment in the cortex, expression of a deep layer neuronal marker at the correct cortical location, and rewiring via transplanted cells with the recipient subcortical target area are important parameters for cell replacement therapy.

It is generally believed that neurons are more sensitive to the hypoxia-ischemia environment than glial cells because they have higher energy demands and only they produce glutamate. In addition, some types of neurons are more vulnerable than others to this type of adverse environment. For example, the hippocampal pyramidal cells of CA1, pyramidal neocortical neurons in layers V and VI, Purikinje cells, and striatal neurons have the highest vulnerability. The HIE in this study was based on the Rice-Vannucci model with relatively brief insults. In this model, there is deep layer-selective neuronal cell death, such as Ctip2-positive deep-layer neurons, in contrast to preserving Cux1-positive superficial-layer neurons from the medial to the lateral side after ischemia (Figures 1D–G) (Vannucci and Vannucci, 1997). This selective vulnerability may depend on the difference between early-developed deep-layer neurons and late-developed superficial-layer neurons (Molyneaux et al., 2007). However, cortical layer II/III neurogenesis is completed by embryonic day 18.3, which can explain why the selective vulnerability in our model does not correlate with the timing of neuronal development because we created the HIE model on postnatal day 2 (Clancy et al., 2001). To summarize these points, the HIE mice at postnatal day 2 in our study were the appropriate model for making a deep layer selective lesion, a laminar necrosis model. This model is suitable for evaluation of region-specific cell replacement, neural network reconstruction, and motor functional recovery after neural stem cell-transplantation.

In this study, although we targeted the transplant to the cortical deep layer, some cells migrated to another cortical area or leaked to the subcallosal region, i.e., striatum or hippocampus unexpectedly. Interestingly, engrafted and leaked NPCs into the hippocampus differentiated to pyramidal shaped-glutamatergic neurons with apical dendrites in the pyramidal layer in the CA1 region (Figures 4M–P). The peak of CA1 development in mice is embryonic day 14.8, which is close to that of the cortical layer V development (day 14.2) when compared to that of cortical layer II/III (day 16.9) (Clancy et al., 2001). This fact could explain why our transplanted NPCs were likely to develop into not only cortical layer V neurons but also into hippocampal pyramidal neurons.

Two established motor tasks, namely the Rotarod test and the Beam walking test, were carried out in this study. The Rotarod test showed that transplanted animals significantly stayed longer on the rotating rod (30–35 s) than vehicle-transplanted animals (20 s). A review of the literature reveals that a 20–30% behavioral improvement after ischemic injury is consistently considered a robust therapeutic benefit (Borlongan et al., 1998, 2004, 2005; Chen et al., 2001). One explanation for this functional recovery might be due to regeneration of the neuronal networks although the function of the engrafted cells and axonal connectivity have not been demonstrated yet. On the other hand, we cannot exclude the possibility that functional recovery was mediated by a neuroprotective effect of the engrafted neural stem cells. In fact, NSCs or NPCs are also thought to provide neuroprotective support by expressing various neurotrophic factors, such as BDNF, GDNF, or NT3 (Park et al., 2002; Buhnemann et al., 2006; Daadi et al., 2009, 2010). This neurotrophic effect could contribute to cell survival, further neuronal differentiation, and significant axonal sprouting (Park et al., 2002; Yasuhara et al., 2008; Daadi et al., 2010).

One would not predict adequate engraftment if the NPCs are transplanted into an ischemic core lesion because this area is not an ideal “niche” i.e., it does not possess a significant blood supply or growth and neurotrophic factors that promote cell survival, neuronal maturation, and axonal sprouting. On the other hand, in ischemic penumbra lesions, increased vascularization within a few days after stroke is associated with neurological recovery and offers another potential target for cell therapy (Krupinski et al., 1993; Senior, 2001; Wei et al., 2001). Because the HIE model used in our study revealed that the ischemic core lesion was located relatively on the lateral side near the middle cerebral artery, the location of cell transplantation was expected to target the boundary adjacent to the penumbra although we cannot confirm this location when transplanting cells (Figures 1A, 4A). Similarly, healthy engraftment could not be confirmed in the cells that were injected into the ischemic core lesion.

In summary, our data show that ES-NPCs transplanted into the injured cortex of an HIE model mouse are capable of differentiation to pyramidal neurons with axonal growth toward the subcortical target area and that these grafted cells might be able to promote motor functional recovery based on the morphological evidence demonstrated in this study. Although we have not demonstrated direct functional recovery due to the graft, these findings demonstrated the possibility of target-specific innervation in injured brain. Even though HIE is an important cause of perinatal mortality and permanent neurological morbidities, including CP, there is no definite treatment for HIE (Ferriero, 2004). Cell replacement therapy for HIE using neural stem cells could be a critical part of a novel therapeutic strategy although we do not know the detailed mechanism of motor functional recovery after NPCs engraftment.

### Conflict of interest statement

The authors declare that the research was conducted in the absence of any commercial or financial relationships that could be construed as a potential conflict of interest.
